# Supplementary material for: Concerning the stability of seawater electrolysis: a corrosion mechanism study of halide on Ni-based anode
Source: Nat Commun. 2023 Aug 10;14:4822. doi: 10.1038/s41467-023-40563-9 (PMC10415325; doi:10.1038/s41467-023-40563-9)
Supplement: Supplementary file 1 — Supplementary Information [file 41467_2023_40563_MOESM1_ESM.pdf]

Concerning the stability of seawater electrolysis: a corrosion mechanism study of halide on Ni-based anode

Sixie Zhang[1,2,3‡], Yunan Wang[1,2,3‡], Shuyu Li[3,4], Zhongfeng Wang[1,2,3], Haocheng Chen[1,2], Li Yi[1,2], Xu Chen[1,2], Qihao Yang[1,2], Wenwen Xu[1,2]*, Aiying Wang[3,4], and Zhiyi Lu[1,2,3]*

1. Key Laboratory of Advanced Fuel Cells and Electrolyzers Technology of Zhejiang Province, Ningbo Institute of Materials Technology and Engineering, Chinese Academy of Sciences, Ningbo 315201, Zhejiang, P. R. China.

2. Qianwan institute of CNITECH, Ningbo 315201, Zhejiang, P. R. China.

3. University of Chinese Academy of Sciences, Beijing 100049, P. R. China.

4. Key Laboratory of Marine Materials and Related Technologies, Ningbo Institute of Materials Technology and Engineering, Chinese Academy of Sciences, Ningbo 315201, Zhejiang, P. R. China.

Table of contents

Glossary..3

Supplementary Figures..4

Supplementary Tables..36

Supplementary References…………………………………………………………...40

Glossary[1,2]

Corrosion

The chemical or electrochemical reaction between a material and its environment that produces a deterioration of the material and its properties.

Passivation

The changing of a chemically active surface of a metal to a much less reactive state.

Passive film (layer)

An ultra-thin film (generally metal oxide) on the surface of the metal, which acts as a barrier to reduce the corrosion rate.

Pitting

Localized corrosion of a metal surface, confined to a point or small area, which takes the form of cavities.

Pit

A type of corrosion crater caused by pitting corrosion.

Re-passivation

The process where actively corroding pits stop pitting, accompanied by the formation of passive film in the pitting area.

Film breakdown potential (E_b)

The positive potential limit of stability of the passive film, above which local sites of the passive film begin pitting.

Pitting potential (E_{pit})

A threshold potential that is obtained in correspondence to an abrupt increase of current density, denotes the propagation and growth of stable pits.

Supplementary Figures

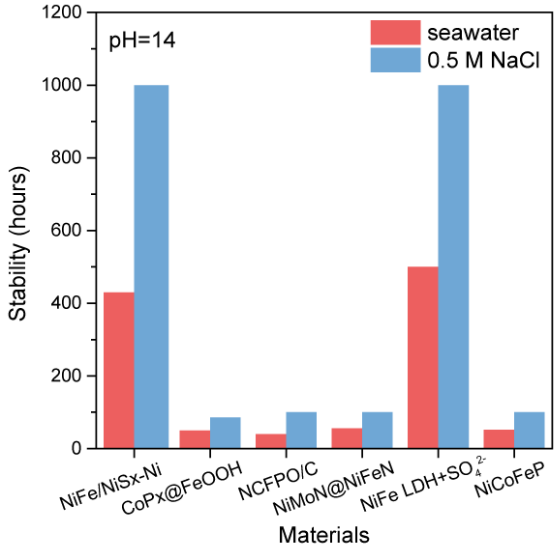

Supplementary Fig.1 Stability comparison of reported anodes[3-8] in 1 M NaOH + seawater and 1 M NaOH + 0.5 M NaCl electrolytes, respectively. All these anodes demonstrated the inferior stability in 1 M NaOH + seawater than that in 1 M NaOH + 0.5 M NaCl.

All these stability data for anodes running in 1 M NaOH + seawater and 1 M NaOH + 0.5 M NaCl were taken under the same voltage difference.

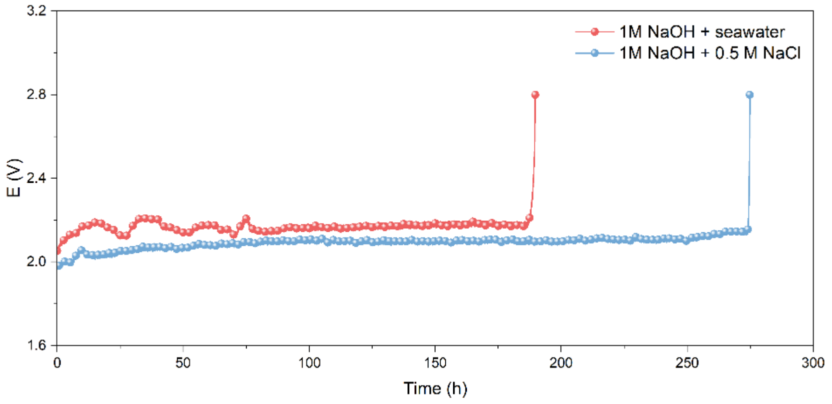

Supplementary Fig.2 Stability comparison of NiFe-H at a current density of 400 mA cm^{-2} in 1 M NaOH + seawater and 1 M NaOH + 0.5 M NaCl electrolytes, respectively.

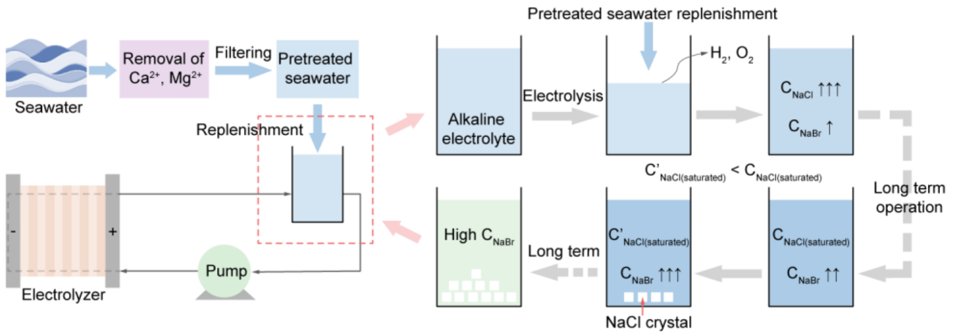

Supplementary Fig.3 Schematic diagram of the accumulation of halides in the electrolyte with the replenishment of seawater during seawater electrolysis process.

The calculation of the accumulated Br^- after one-year seawater electrolysis with the continuous supplement of seawater:

For industrial 1 kW seawater electrolysis equipment[7] applied to hydrogen production, the electrode area and the operating current density of the anode is 1960 cm^2 and 500 mA cm^{-2}, respectively. The volume of the electrolyte inside the cell (half side of the electrolyzer, V_{cell}) is 3 L. The initial concentration of Br^- ($[Br^-]$) is 0.53 mM. The molar quantity of water consumption (n_{H_2O}) is calculated by Supplementary equation (1), where Q is the total amount of charge, F is the Faraday constant, z is the number of charges transferred, and the Faradic efficiency is assumed to be 100%. The final concentration of Br^- (C_{Br^-}) is obtained from Supplementary equation (2), where the t is one year, $V_{seawater}$ is the volume of seawater feeding per second, which is calculated by Supplementary equation (3). The result shows that C_{Br^-} will reaches 0.5 M after one-year operation

$$n_{H_2O} = \frac{Q}{z \times F} \quad (1)$$

$$C_{Br^-} = [Br^-] + [Br^-] \times \frac{V_{seawater}}{V_{cell}} \times t \quad (2)$$

$$V_{seawater} = \frac{n_{H_2O} \times M_{H_2O}}{\rho_{H_2O}} \quad (3)$$

In the practical operation conditions, with the replenishment of seawater, NaCl will definitely accumulate to the saturated concentration (~5 M), which is fatal to the electrodes. For this reason, our team has reported that the increase of NaOH concentration can effectively reduce the saturated NaCl content in the electrolyte.[7] Due

to the common-ion effect, the solubility of NaCl will significantly decrease from ~5 M to ~2.5 M when the concentration of NaOH increase to 6 M. As the decrease of Cl^- content and corrosion-inhibiting effect of OH^-, the corrosion of electrode can be alleviated, which is conducive to the stability of the anode. Thus, electrodes operating in 6M NaOH + seawater electrolyte must be the future trend of practical application.

Notably, the concentration of Br^- will also increase with the continuous addition of seawater. More importantly, as the solubility of NaBr is higher than NaCl in water[9], it will cause the decrease of saturation solubility of NaCl in electrolytes containing 6M NaOH. Therefore, with the reaction time prolong, the concentration of Cl^- will be less than 2.5 M in 6M NaOH electrolyte, while the concentration of Br^- will continuously increase (Supplementary Fig.3). It is predictable that after some time, Br-induced corrosion of anodes will dominate.

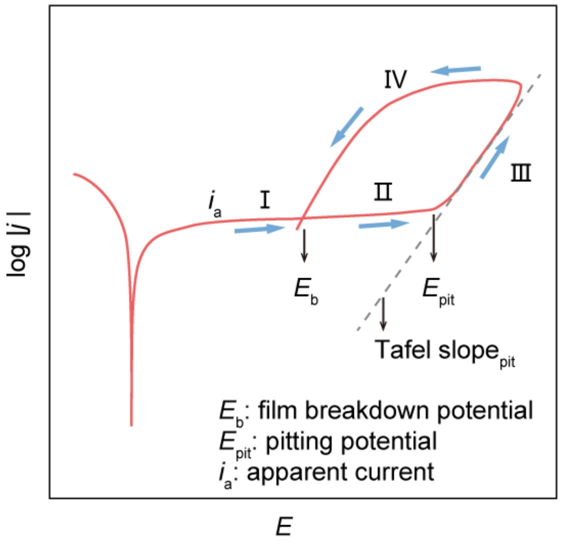

Supplementary Fig.4 Schematic representation of a generic anodic CPC.

Supplementary Fig.4 displays a standard anodic CPC with a hysteresis loop measured in the alkaline electrolyte, which can be divided into four regions: I) passive dominant state, II) metastable pitting corrosion state, III) stable pit growth state, and IV) re-passivation state[2,10-14]. In the passive dominant stage, a passive layer (i.e., metal oxide) forms on the metal surface and becomes thicker, accompanied by a slower pitting process. Due to the low reactivity of the passive layer, the apparent current, which equals the passivation current subtracted pitting current, will remain at a quite low value. When the voltage exceeds the film breakdown potential (E_b) (i.e., metastable pitting corrosion state), pits start to nucleate at defective sites of the passive layer. In the meantime, some pitting areas may also re-passivate, resulting in the halt of the pits growing. The anode will maintain an unstable pitting process until the voltage is higher than the threshold potential E_{pit}, where the anode enters the stable pit growth state. In the stable pit growth stage, the pits grow rapidly with a remarkable increase in current, causing severe damage to the anode. As the voltage decreases, the pitting dissolution of the metal ceases and re-passivates quickly, leading to the recovery of the passive film and the appearance of a hysteresis loop in the CPC.

Notably, several crucial characteristic parameters can be obtained from the curve: i) E_b, above which local sites of the passive film begin pitting; ii) E_{pit}, a threshold potential

that is obtained in correspondence to an abrupt increase of current density, denotes the propagation and growth of stable pits; iii) Tafel slope of pitting (Tafel slope $_{pit}$), which denotes the linear relationship between logarithm of current density and the potential in the region where the potential was larger than E_{pit}, and can reflect the pitting kinetic of the anode.

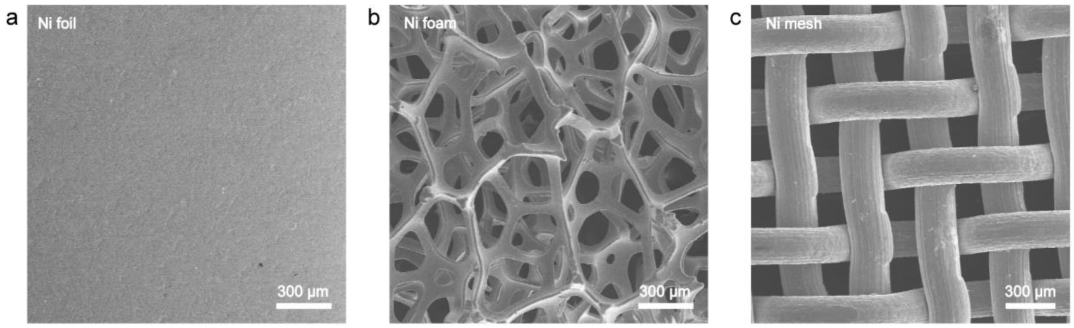

Supplementary Fig.5 a, **b**, **c** Morphologies of Ni foil, Ni foam, and Ni mesh, respectively.

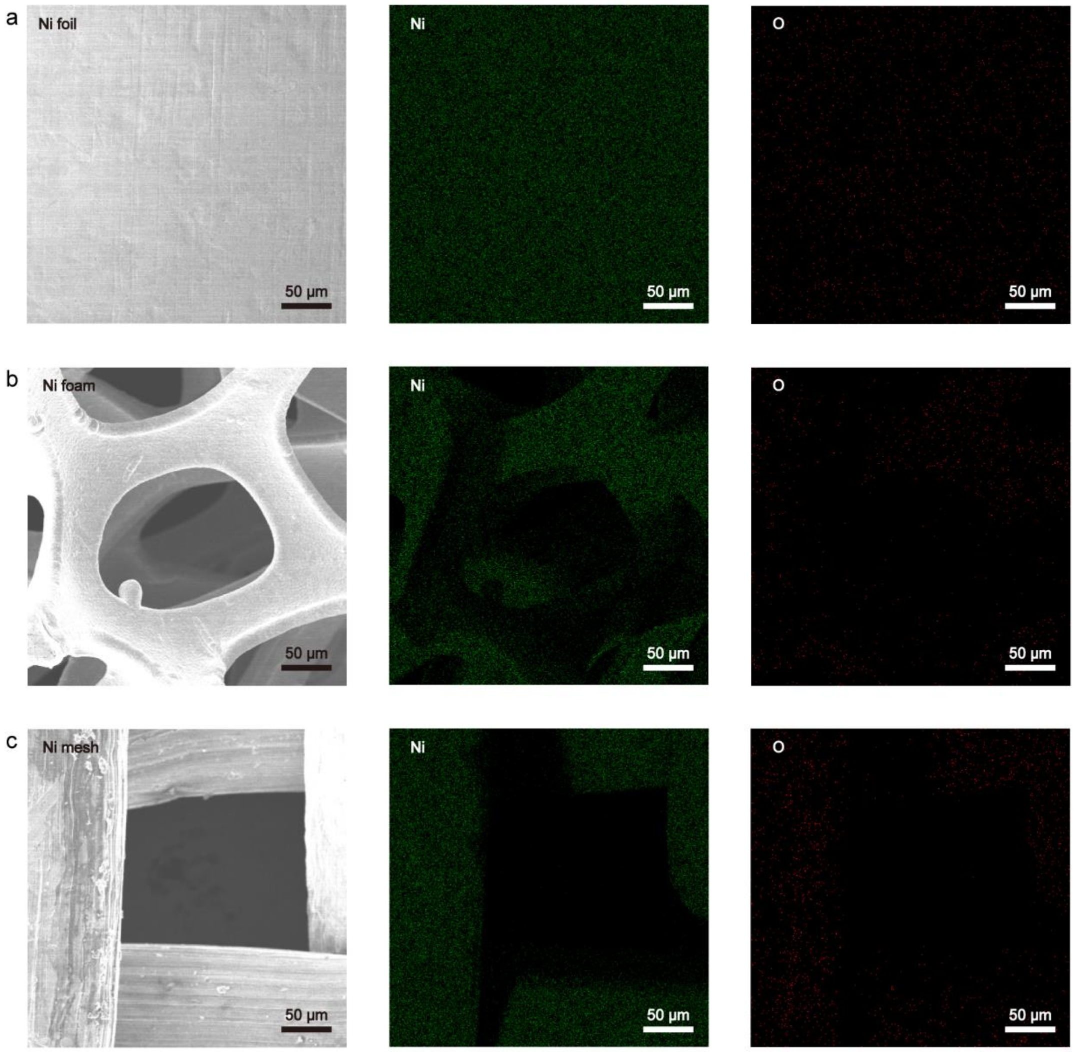

Supplementary Fig.6 a, **b**, **c** Morphologies and corresponding SEM-EDX mappings of Ni foil, Ni foam, and Ni mesh, respectively. The globally homogeneous distribution of Ni (green) and O (red) species can be observed, indicating the spontaneously formed oxide passive film on the surface.

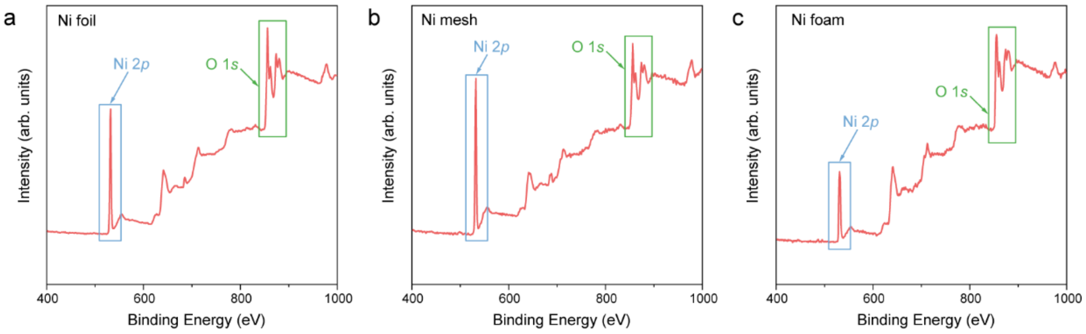

Supplementary Fig.7 a, **b**, **c** XPS surveys of Ni foil, Ni mesh, and Ni foam, respectively.

The results demonstrated that the Ni substrates were composed of Ni and O, which was consistent with the SEM-EDX mappings.

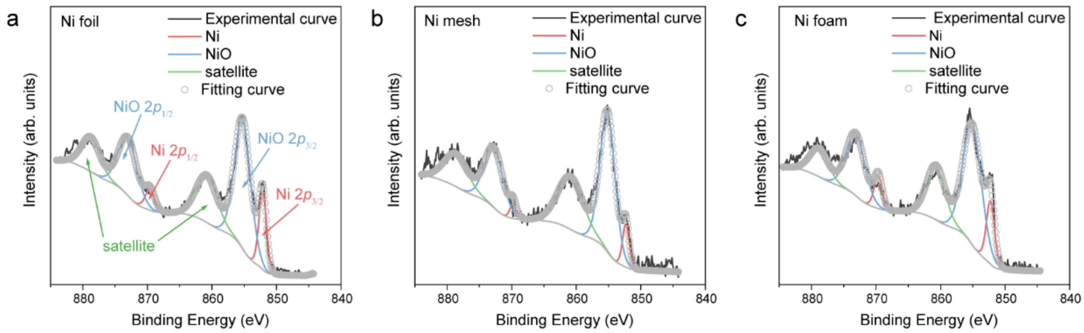

Supplementary Fig.8 a, **b**, **c** High-resolution Ni 2*p* spectrum of Ni foil, Ni mesh, and Ni foam. The deconvoluted spectrums illustrated that the coexistence of Ni and NiO.[15,16]

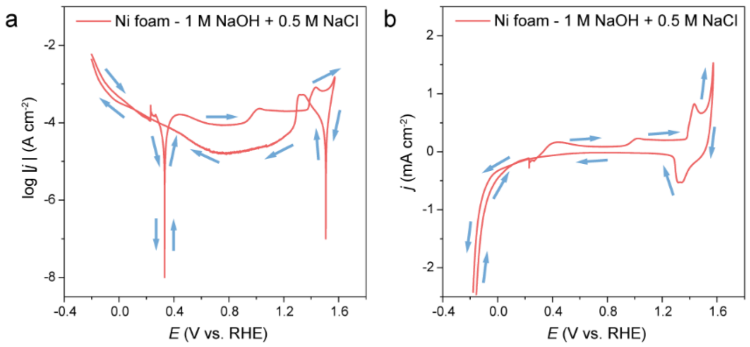

Supplementary Fig.9 a The CPC of Ni foam measured in 1 M NaOH + 0.5 M NaCl electrolyte. **b** The corresponding Cyclic voltammetry (CV) curve. The scan rate was 10 mV s^{-1}.

Notably, the disappearance of the hysteresis loop in the CPC can be observed in this solution system, which may be attributed to the signal interference from OER. Due to the high concentration of OH^-, more OH^- could be adsorbed on the anode surface, leading to the occurrence of OER at low voltage. Consequently, the current generated by corrosion was hardly to identify in CPC. In addition, the Ni^{2+}/Ni^{3+} redox peak can also be observed in CV curve, which can also interfere the pitting signal. Thus, the electrolyte with high concentration of OH^- was not suitable for the CPC measurement.

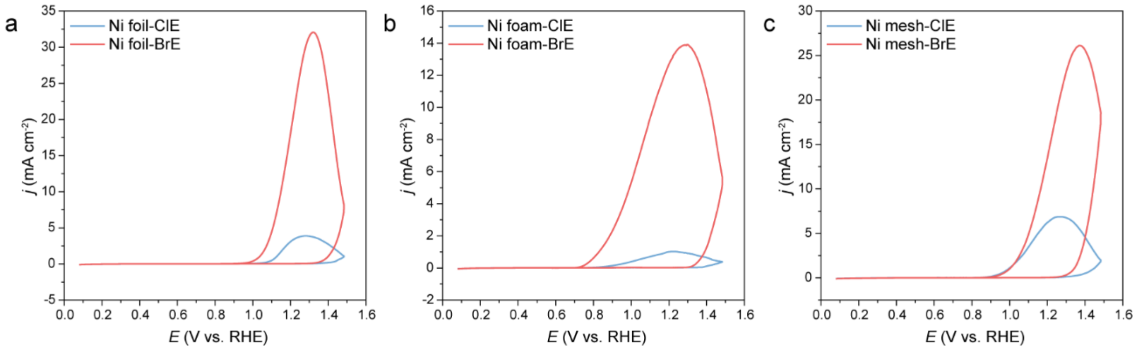

Supplementary Fig.10 a, **b**, **c** The corresponding CV curves of Ni foil, Ni foam, and Ni mesh measured in ClE and BrE, and the scan rate was 1 mV s^{-1}.

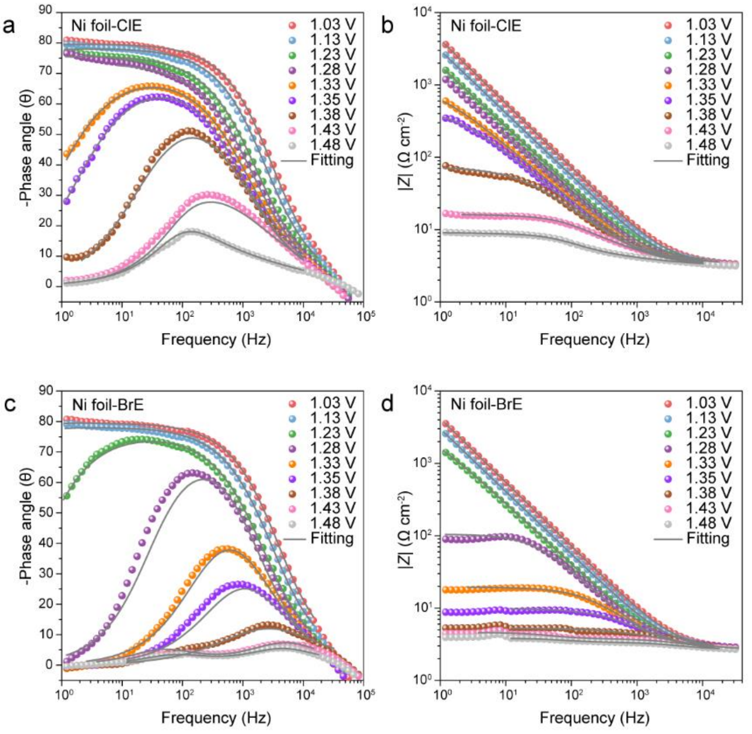

Supplementary Fig.11 **a**, **b**, **c**, **d** Bode-phase and Bode-amplitude plots of Ni foil at different potentials in ClE and BrE, respectively. The solid line was the fitted value.

With the occurrence of corrosion, the low-frequency inductive behavior could be observed in the Nyquist plots, which may be attributed to the metal dissolution process[17,18] or the adsorption of intermediate species.[19] Besides, the corrosion process also contained the diffusion of reactants and products, increasing the complexity of the impedance analysis. We have tried a variety of Equivalent circuit models (ECs) to fit the impedance spectra. Unfortunately, none of them fitted the low-frequency part of impedance spectra pretty well. The good thing is that ECs used here were well fitted in the mid-high frequency region of impedance spectra. This region corresponded to the $Ni^0 \rightarrow Ni^{2+}$ transition, denoting the corrosion process. Thus, we believed it is reasonable to use such simple ECs to roughly assess the corrosion process and draw the conclusion that Ni foil was easier to be corroded by Br^- than Cl^-.

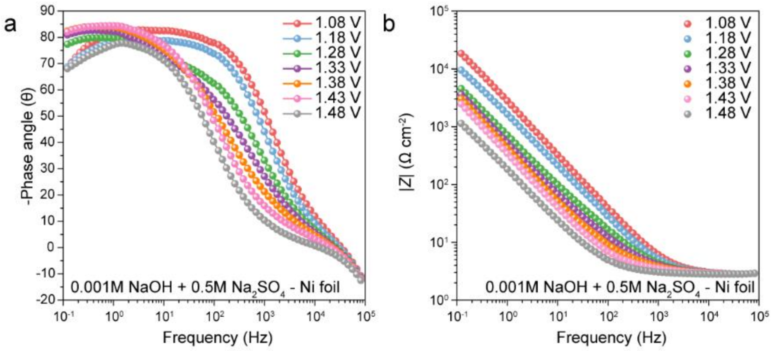

Supplementary Fig.12 a, **b** Bode plots of Ni foil at different potentials in 0.001 M NaOH + 0.5 M Na_2SO_4 electrolyte. Compared with the Bode plots measured in ClE and BrE, the phase angle at the middle-frequency region maintained a high value in Na_2SO_4 electrolyte with the change of potential, indicating the decrease of phase angle at the middle-frequency region in ClE and BrE was caused by corrosion.

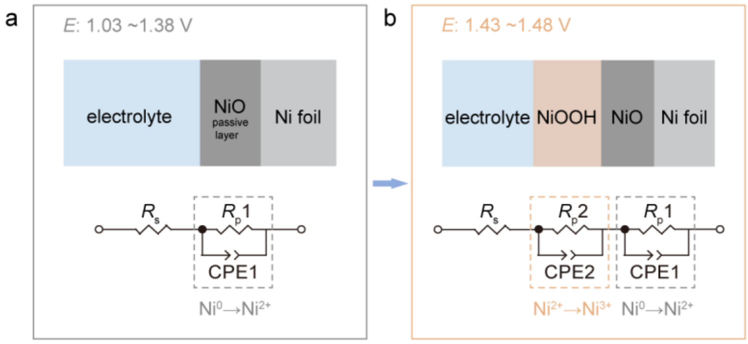

Supplementary Fig.13 a, **b** Equivalent circuit models for pitting corrosion of Ni foil in different potential intervals, for 1.03 V to 1.38 V, for 1.43 V to 1.48 V.

In the equivalent circuit models, the R_s represented the solution resistance. $CPER_p$ loops represented the dielectric property and resistivity of the interface reaction with electron transfer at the middle frequency, where $CPE1R_p1$ loop denoted the electrooxidation of Ni^0 to Ni^{2+} at the pit area due to the pitting corrosion of Ni electrodes by halide, and $CPE2R_p2$ loop denoted the electrooxidation of Ni^{2+} to Ni^{3+} at the potential larger than 1.43 V (the threshold for Ni^{2+}/Ni^{3+} oxidation).

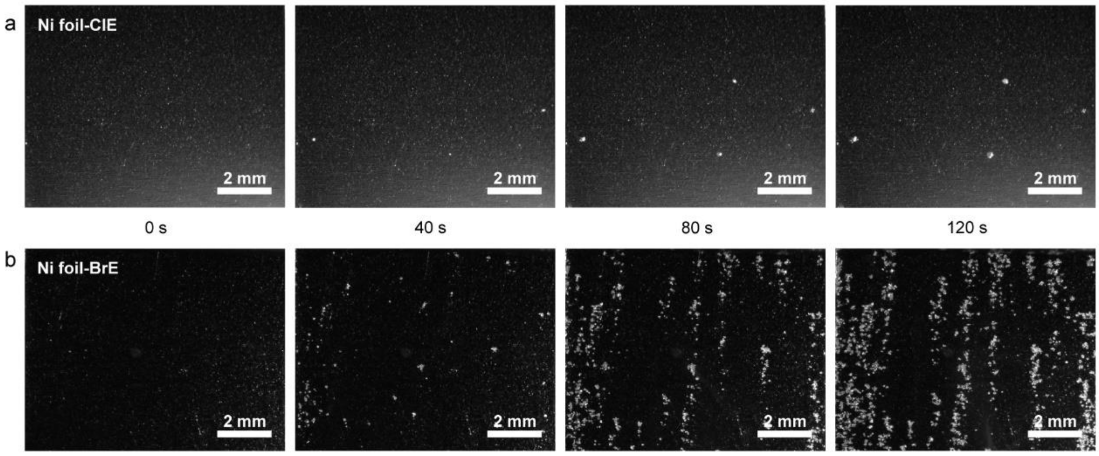

Supplementary Fig.14 a, **b** In situ optical microscopy photos of Ni foil corroded by Cl^- and Br^-, respectively.

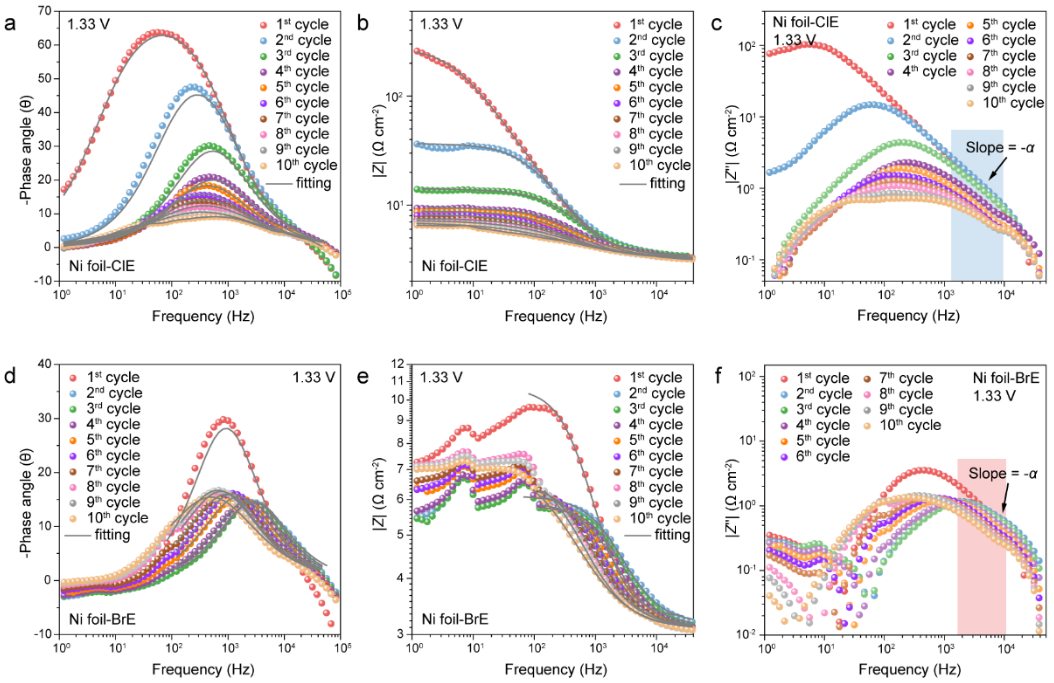

Supplementary Fig.15 a, **b**, **d**, **e** Time-dependent Bode-phase and Bode-amplitude plots of Ni foil in ClE and BrE, respectively. **e**, **f** Corresponding imaginary part of the impedance-frequency plots.

The time-dependent operando EIS curves of Ni foil were measured in ClE and BrE with the applied voltage of 1.33 V vs. RHE, and all these curves (10 cycles) were measured continuously one after another. As shown in Supplementary Fig.15a and 15d, the phase angle at the middle-frequency region in BrE was smaller than that in ClE, indicating that Br^- was more aggressive than Cl^- for Ni foil. As the number of cycles increased, the phase angles for Ni foil in ClE decreased gradually, while these in BrE were maintained, which was consistent with the potentiostatic polarization curves.

Furthermore, the surface morphology change of the anode can be reflected by the variation of constant phase element (CPE) exponent (α) values. Typically, the CPE is considered as a mathematical expression (Equation (S5)) of a special case of time-constant dispersion[20] that is attributed to surface inhomogeneity, roughness or fractal geometry, and electrode porosity, etc.[21]

$$Z_{\mathrm{CPE}} = \frac{1}{(\mathrm{j}\omega)^{\alpha} Q} \qquad \text{(S5)}$$

Where ω is angular frequency (rad/s), Q is a coefficient (with dimensions F cm^2 s$^{-(1-\alpha)}$), and α is the dispersion coefficient, which can reflect the surface geometry of

the electrode.[21,22] Notably, the CPE serves as an ideal capacitance when α =1. When 0.5 < α < 1, the value of α can reflect the roughness of the electrode surface. A value 0 < α indicates the pore structure of the electrode.

We used the EC shown in Supplementary Fig.13a to quantify the α and investigate the change in the surface morphology of anode during the corrosion process (Supplementary Table 4). However, the fitting curves also did not match very well in the low-frequency region. Thus, in order to obtain an accurate value of α, the graphical method[23,24] was adopted. We plotted the imaginary part of the impedance - frequency plots (Supplementary Fig.15c and 15f), where the slope at high frequency was -α. Therefore, by fitting the slope in the linear part of the high-frequency region, we obtained the variation of α with cycles, and the results were shown in Fig. 2e. As the number of cycles increased, α of Ni foil in ClE decreased gradually to less than 0.5, suggesting the formation of severe pitting pores. In contrast, α of Ni foil in BrE were maintained, indicating that the electrode surface became rough due to corrosion.

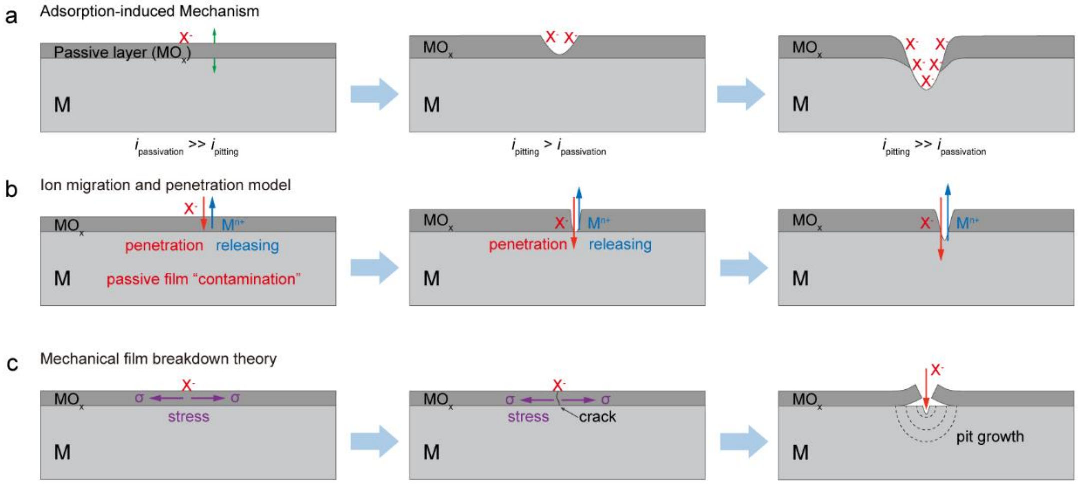

Supplementary Fig.16 a, **b**, **c** Schematic illustrations of three mechanisms for pit initiation, including adsorption-induced mechanism, ion migration and penetration model, and mechanical film breakdown theory.[12,25,26]

(a) Adsorption-induced mechanism. When the voltage is applied to anodes, halide ions will adsorb on the anode surface and then replace the surface oxygen of the passive layer, leading to the formation of a complex with metal ions. Then the complex will dissolve and diffuse into the solution to form the hydroxide. Specifically, the passivation rate is faster than corrosion rate ($i_{passivation} > i_{pitting}$) at the beginning, the passive layer becomes thicker. When the corrosion rate increases and becomes larger than passivation rate ($i_{pitting} > i_{passivation}$), the passive layer will become thinner at the corrosion sites. As the corrosion rate continues to increase and becomes much higher than passivation rate ($i_{pitting} >> i_{passivation}$), the breakage of passive film and the corrosion of the underneath substrate will happen.

(b) Ion migration and penetration model. Due to the influence of the high electric field, halide ions will penetrate and contaminate the oxide film, causing higher electrical and ionic conductivity along the penetration paths. During the penetration process, some vacancies may also form in the passive film. Thereby the rapid release of cation at the film-solution interface or the accumulation of vacancies at the metal-film interface may happen. Finally, the passive film will break down and followed by the corrosion of substrate.

(c) Mechanical film breakdown theory. Some mechanical factors, including

electrostriction stress, blistering, and micro-capillary formation, may lead to the strain on the passive film. At some weak sites, the stress on the film may exceed the mechanical breakdown stress, causing the formation of cracks in the film. Then the film will rupture rapidly and form a channel where the halide ions can pass through to corrode the underneath metal.

Note that the mechanical film breakdown usually caused by mechanical factors, which was less relevant to the halide species. Thus, we consider that the pitting initiation of Ni substrates in ClE and BrE mainly involves a combination of adsorption and permeation mechanisms. Therefore, we conducted the density functional theory simulations to study the adsorption and carried out the nudged elastic band calculations to investigate the diffusion.

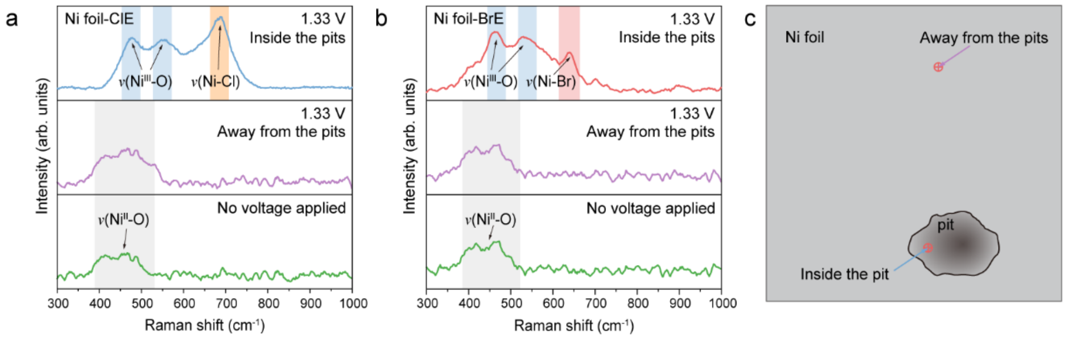

Supplementary Fig.17 a, **b** Quasi in situ potential-dependent Raman spectra of Ni foil in ClE and BrE, respectively. **c** Schematic illustrations of Raman detection location.

As shown in Supplementary Fig.17, the original surface of Ni foil was covered by the passive layer NiO[27], which was consistent with the SEM mapping and XPS results. When the potential reached 1.33 V vs. RHE, *v*(Ni-Cl) (682 cm^{-1}) and *v*(Ni-Br) (630 cm^{-1}) [28] vibration signals could be detected around/inside the pits, while no signal was detected away from the pits, indicating that the localized adsorption of halides enhanced the corrosion process. Notably, the intensity of Ni-Cl vibration was stronger than that of Ni-Br, which was consistent with SEM and SVET results, indicating the high Ni-Cl intermediate concentration inside the pits due to their narrow-deep shapes.

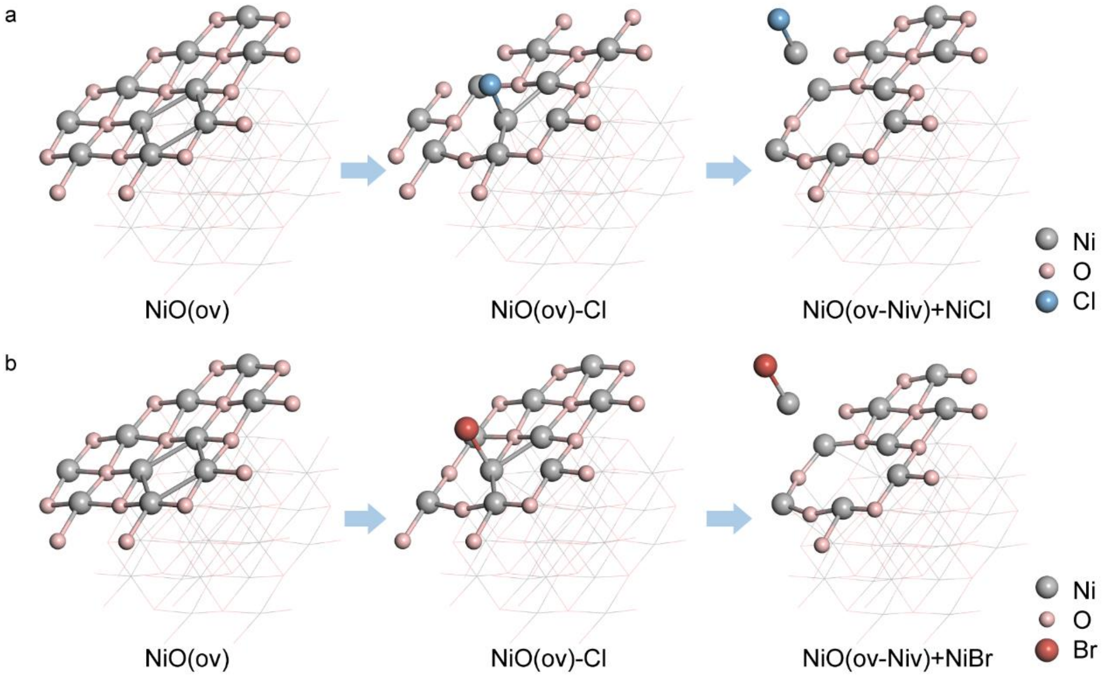

Supplementary Fig.18 a, **b** The pathways of corrosion process of NiO in ClE and BrE, respectively. Red, blue, gray, and pink balls denote Br, Cl, Ni, and O atoms.

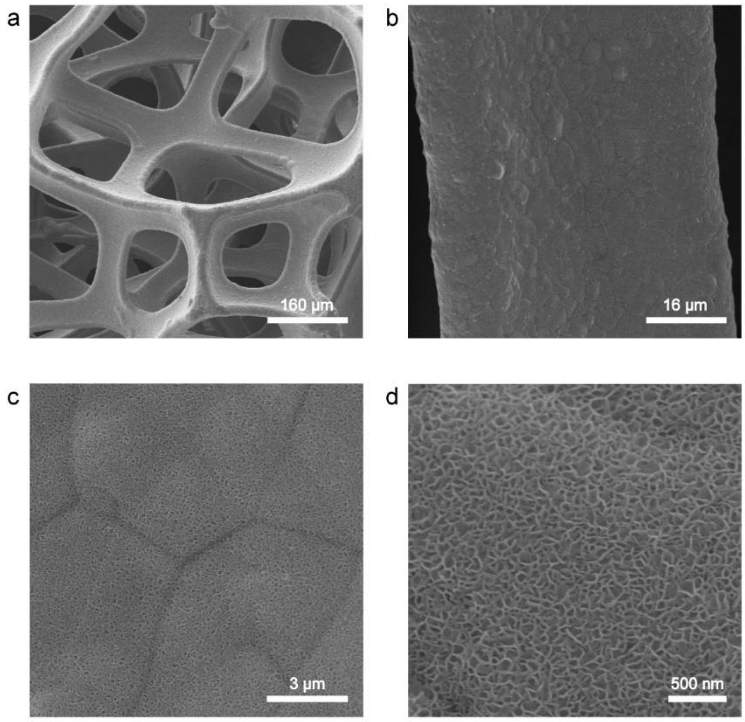

Supplementary Fig.19 Morphologies of NiFe-E.

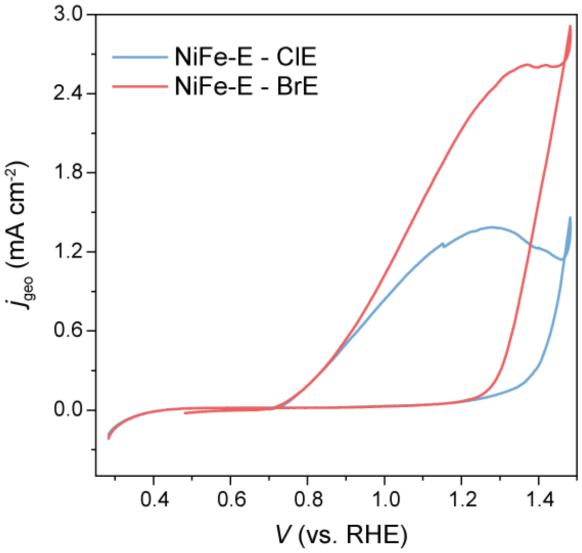

Supplementary Fig.20 The CV curves of NiFe-E measured in ClE and BrE, which was corresponding to the CPC. The scan rate was 10 mV s^{-1}.

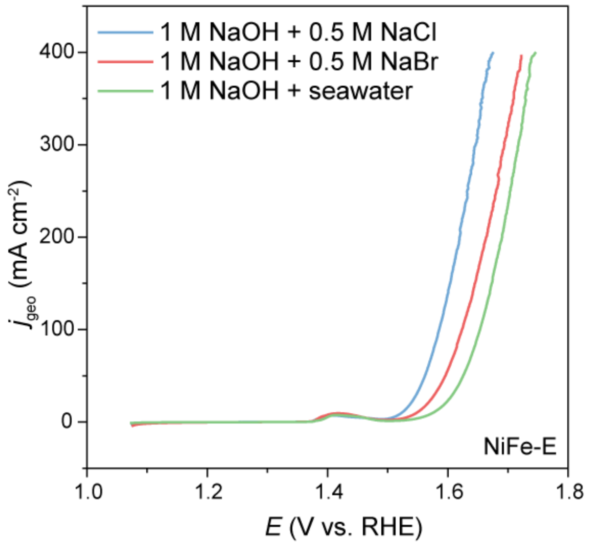

Supplementary Fig.21 The LSV curves of NiFe-E anode measured in 1 M NaOH + 0.5 M NaCl , 1 M NaOH + 0.5 M NaBr, and 1 M NaOH + seawater electrolytes, respectively. The scan rates were 5 mV s^{-1}.

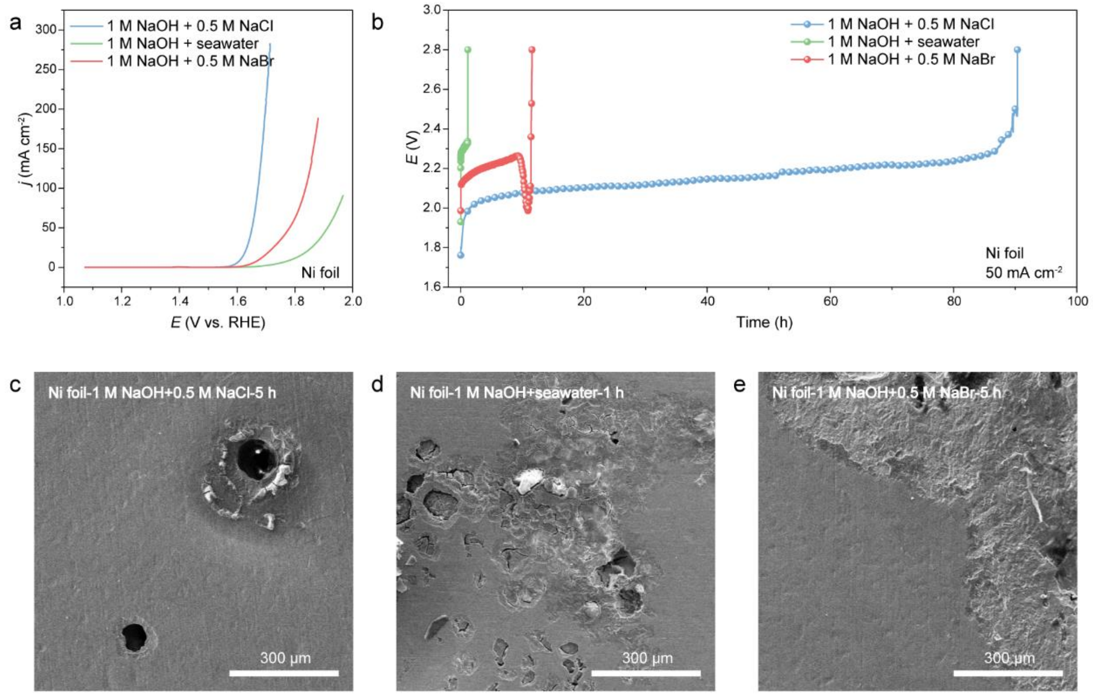

Supplementary Fig.22 a The LSV curves of Ni foil anode measured in 1 M NaOH + 0.5 M NaCl, 1 M NaOH + seawater, and 1 M NaOH + 0.5 M NaBr electrolytes, respectively. The scan rate was 5 mV s^{-1}. **b** The corresponding durability tests of Ni foil at a current density of 50 mA cm^{-2}. **c**, **d**, **e** Corresponding SEM images of Ni foil observed after 5 h operation in 1 M NaOH + 0.5 M NaCl, 1 h operation in 1 M NaOH + seawater, and 5 h operation in 1 M NaOH + 0.5 M NaBr, respectively.

The stability of Ni foil in the 1 M NaOH + seawater at a current density of 50 mA cm^{-2} was obtained to investigate the corrosion behavior of Ni foil in the real seawater (Supplementary Fig. 22). By the comparison of the corrosion behaviors in ClE and BrE, both narrow-deep (ClE) and wide-shallow (BrE) pits were found on Ni foil (Supplementary Fig. 22d), indicating that the Ni substrates exhibited a mixed corrosion behavior in alkaline natural seawater. Thus, the influence of trace Br^- (0.53 mM) on the corrosion is also significant.

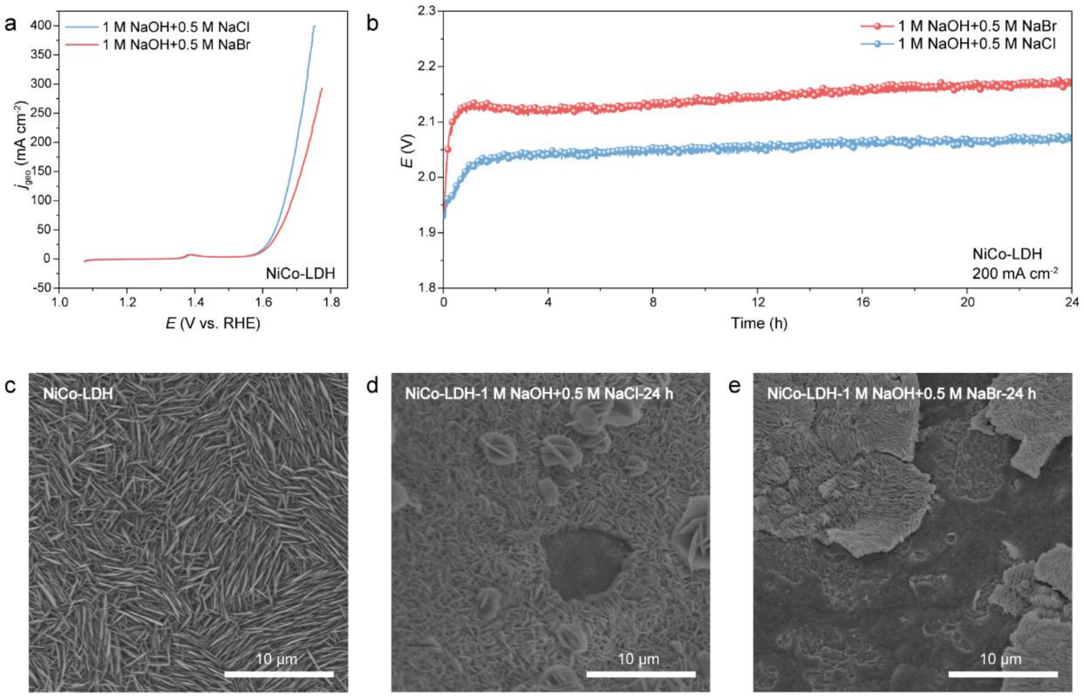

Supplementary Fig.23 a The LSV curves of NiCo-LDH anode measured in 1 M NaOH + 0.5 M NaCl and 1 M NaOH + 0.5 M NaBr electrolytes. The scan rate was 5 mV s^{-1}. **b** The corresponding durability tests of NiCo-LDH at a current density of 200 mA cm^{-2}. **c**, **d**, **e** Corresponding SEM morphologies of original NiCo-LDH and morphologies observed after 24 h operation in 1 M NaOH + 0.5 M NaCl and in 1 M NaOH + 0.5 M NaBr.

We also investigated the corrosion behaviors of the typical OER electrocatalysts including NiCo-LDH nanoarrays/Ni foam (NiCo-LDH) and phosphorus-doped NiFe-LDH nanoarrays/Ni foam (NiFeP) in 1 M NaOH + 0.5 M NaCl and 1 M NaOH + 0.5 M NaBr electrolytes. The results (Supplementary Fig. 23 and 24) revealed that the corrosion behaviors of NiCo LDH and NiFeP in Br^- and Cl^- were comparable to that of the NiFe-E electrode, indicating that Br- corrosion induced spalling of the catalyst layer was a common phenomenon in integrated electrodes (substrate + catalyst on surface).

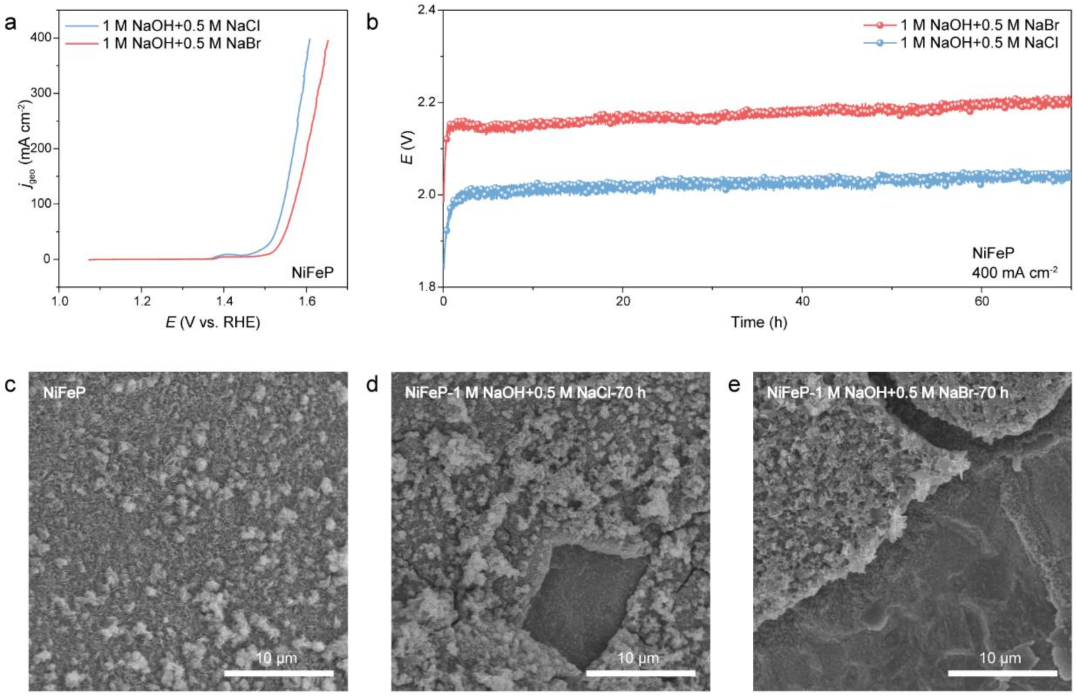

Supplementary Fig.24 a The LSV curves of NiFeP anode measured in 1 M NaOH + 0.5 M NaCl and 1 M NaOH + 0.5 M NaBr electrolytes. The scan rate was 5 mV s^{-1}. **b** The corresponding durability tests of NiFeP at a current density of 400 mA cm^{-2}. **c**, **d**, **e** Corresponding SEM morphologies of original NiFeP and morphologies observed after 70 h operation in 1 M NaOH + 0.5 M NaCl and in 1 M NaOH + 0.5 M NaBr.

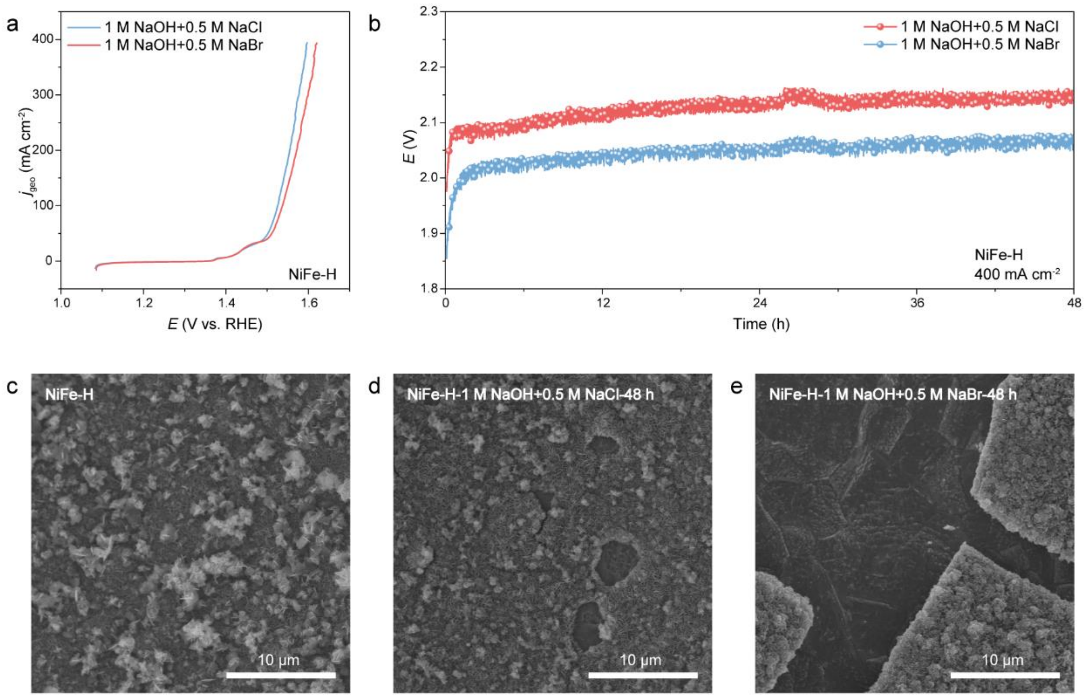

Supplementary Fig.25 a The LSV curves of NiFe-H anode measured in 1 M NaOH + 0.5 M NaCl and 1 M NaOH + 0.5 M NaBr electrolytes. The scan rates were 5 mV s^{-1}. **b** The corresponding durability tests of NiFe-H at a current density of 400 mA cm^{-2}. **c**, **d**, **e** Corresponding SEM morphologies of original NiFe-H and morphologies observed after 70 h operation in 1 M NaOH + 0.5 M NaCl and in 1 M NaOH + 0.5 M NaBr.

We synthesized the NiFe-LDH nanoarrays electrode with different morphologies by electrodeposition (NiFe-E, Supplementary Fig. 19) and hydrothermal method (NiFe-H, Supplementary Fig. 25c). Their corrosion behaviors were studied in 1 M NaOH + 0.5 M NaCl and 1 M NaOH + 0.5 M NaBr electrolytes. These results illustrated that both NiFe-E and NiFe-H exhibited similar corrosion behaviors in the same electrolyte, where Cl^- led to the formation of pits and Br^- caused the exfoliation of catalyst layer (Fig. 5d and 5e, Supplementary Fig. 25d and 25e).

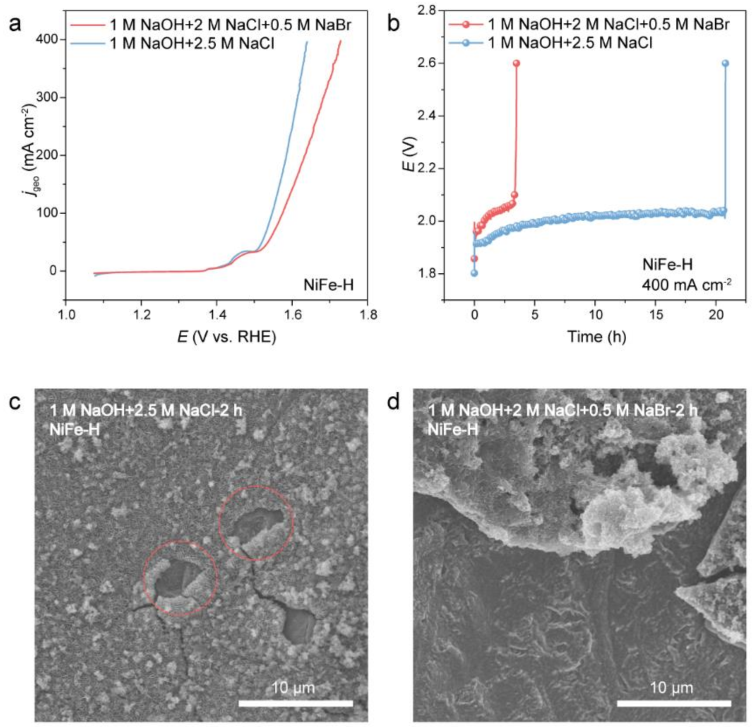

Supplementary Fig.26 a LSV curves of NiFe-H measured in 1 M NaOH + 2.5 M NaCl and 1 M NaOH + 2 M NaCl + 0.5 M NaBr electrolytes, respectively. The scan rates were 5 mV s^{-1}. **b** The corresponding durability of NiFe-H at a current density of 400 mA cm^{-2}. **c**, **d** Corresponding morphology of NiFe-H observed after 2 h operations.

As the replenishment of seawater, in addition to the increase of Br^- content, the concentrations of Cl^- and Br^- would also increase, which was harmful to the anodes and might affect their corrosion behaviors (see details in Supplementary Fig. 3). Therefore, the corrosion behaviors of anodes in high salt concentrations were also investigated. We found that Br^- had a non-negligible influence on the corrosion behavior of anode even if the Cl^- concentration was increased by 5-fold (2.5 M). As shown in Supplementary Fig. 26, the lifetime of NiFe-H anode was drastically reduced in 1 M NaOH + 2 M NaCl + 0.5 M NaBr electrolyte, indicating the harmful corrosion effect of Br^-. The morphologies of NiFe-H anodes after the 2 h durability test in these two electrolytes were observed. In 1 M NaOH + 2.5 M NaCl electrolyte, only some pits appeared on the surface of anode (Supplementary Fig. 26c), while in 1 M NaOH + 2 M NaCl + 0.5 M NaBr electrolyte, the spalling of catalyst layer accompanied by some pits

on the Ni substrate can be observed (Supplementary Fig. 26d). Thus, even at high salt concentrations, the corrosion behavior of anode in chloride and bromide was similar to that of low salt concentrations.

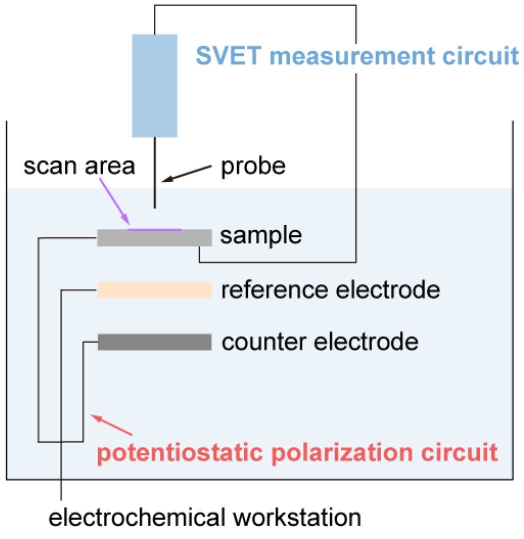

Supplementary Fig.27 Schematic diagram of SVET measurements.

There were two current circuits in the test, one was the circuit for potentiostatic polarization tests applied on the sample, and the other was the circuit for SVET measurements (Supplementary Fig.27). The potentiostatic polarization test was continually operated with an applied voltage of 1.3 V vs. RHE. The SVET measurements were conducted simultaneously with the potentiostatic polarization test.

Theoretically, the anodic current should be equal to the cathodic current in the SVET maps. However, our SVET maps showed a huge unbalance between the total anodic and cathodic ionic currents. After reviewing the relevant literatures, we summarized two main reasons that may cause the unbalance. 1) The scan area was 4 mm × 4 mm, while the area of sample subjected to potentiostatic polarization was 1 cm × 2 cm. The total anodic and cathodic ionic currents were balanced over the entire sample, while they might be unbalanced in the local area (i.e., scan area). 2) Due to the time required to complete a map, the potential signals obtained were not simultaneous. The current distribution on the scan area might change during the scanning[29], resulting in huge unbalance.

Supplementary Tables

Supplementary Table 1. Content of anions in seawater measured by ion chromatography.

Species	Cl^-	Br^-
Concentration (mol L^{-1})	0.41	0.00053

Seawater used in this study was taken from west coast of the East China sea in Ningbo City, Zhejiang Province, China, located at 29°45'13"N and 121°55'27"E.

Supplementary Table 2. E_b, E_{pit}, and Tafel slopes$_{pit}$ values of three Ni substrates tested in ClE and BrE.

Parameters	E_b (V vs. RHE)		E_{pit} (V vs. RHE)		Tafel slopes$_{pit}$ (mV dec^{-1})	
Substrates	ClE	BrE	ClE	BrE	ClE	BrE
Ni foil	0.89736	0.80936	1.29136	1.25936	137.44	80.12
Ni mesh	0.81536	0.79436	1.23336	1.19536	143.17	85.20
Ni foam	0.75736	0.69836	1.372	1.293	60.60	55.40

Supplementary Table 3. Fitting parameters for potential-dependent impedance date of Ni foil in ClE and BrE.

Electrolytes	E (V vs. RHE)	R_s (Ω)	R_p1 (Ω)	CPE1-Q (S s^n cm^{-2})	CPE1-α	R_p2 (Ω)	CPE2-Q (S s^n cm^{-2})	CPE2-α
ClE	1.03	3.437	$1.091*10^6$	$4.635*10^{-5}$	0.886	-	-	-
	1.13	3.406	323040	$6.697*10^{-5}$	0.871	-	-	-
	1.23	3.437	285200	$1.154*10^{-4}$	0.844	-	-	-
	1.28	3.355	$7.51*10^{10}$	$1.724*10^{-4}$	0.815	-	-	-
	1.33	3.299	1103	$2.499*10^{-4}$	0.791	-	-	-
	1.35	3.314	493.5	$3.026*10^{-4}$	0.784	-	-	-
	1.38	3.236	70.53	$3.441*10^{-4}$	0.773	-	-	-
	1.43	3.008	8.136	$1.274*10^{-3}$	0.641	5.221	$4.252*10^{-4}$	0.961
	1.48	3.004	3.059	$8.251*10^{-3}$	0.491	3.136	$9.139*10^{-4}$	0.945
BrE	1.03	2.819	$1.469*10^6$	$4.718*10^{-5}$	0.884	-	-	-
	1.13	2.833	309790	$6.714*10^{-5}$	0.869	-	-	-
	1.23	2.861	3973	$1.093*10^{-4}$	0.848	-	-	-
	1.28	2.929	102.6	$1.007*10^{-4}$	0.878	-	-	-
	1.33	2.852	16.66	$1.638*10^{-4}$	0.833	-	-	-
	1.35	2.784	7.074	$2.282*10^{-4}$	0.801	-	-	-
	1.38	2.607	2.696	$6.651*10^{-4}$	0.685	-	-	-
	1.43	2.712	1.05	$1.911*10^{-4}$	0.834	0.842	$7.819*10^{-3}$	0.896
	1.48	2.664	0.706	$2.512*10^{-4}$	0.849	0.471	$9.588*10^{-3}$	0.889

Supplementary Table 4. Fitting parameters for time-dependent impedance date of Ni foil in ClE and BrE.

Electrolytes	cycle	R_s (Ω)	R_p1 (Ω)	CPE1-Q (S s^n cm^{-2})	CPE1-α
ClE	1	3.431	280.5	$2.106*10^{-4}$	0.82911
	2	3.464	33.31	$1.699*10^{-4}$	0.84807
	3	3.399	10.67	$2.512*10^{-4}$	0.81664
	4	3.282	6.221	$5.699*10^{-4}$	0.75943
	5	3.263	5.582	$8.161*10^{-4}$	0.74420
	6	3.232	4.991	$1.494*10^{-3}$	0.69406
	7	3.217	4.376	$1.852*10^{-3}$	0.67383
	8	3.172	4.11	$2.971*10^{-3}$	0.62058
	9	3.114	3.833	$5.559*10^{-3}$	0.55261
	10	3.046	3.763	$8.522*10^{-3}$	0.49709
BrE	1	3.269	7.412	$8.881*10^{-5}$	0.91368
	2	3.172	2.95	$1.112*10^{-4}$	0.85326
	3	3.111	2.916	$1.321*10^{-4}$	0.84391
	4	3.084	3.12	$1.994*10^{-4}$	0.81894
	5	3.079	3.539	$2.793*10^{-4}$	0.80501
	6	3.087	3.741	$3.147*10^{-4}$	0.80729
	7	3.106	3.949	$4.313*10^{-4}$	0.79035
	8	3.09	4.476	$5.462*10^{-4}$	0.77844
	9	3.088	4.404	$5.748*10^{-4}$	0.77954
	10	3.095	4.146	$8.386*10^{-4}$	0.75296

Supplementary References

1 Norman, E. H. NACE glossary of corrosion terms. *Mater. Prot.* **4**, 79 (1965).

2 Cramer, S. D. & Covino, B. S. *ASM Handbook: v. 13A Corrosion: Fundamentals, Testing, and Protection* (ASM International, 2003).

3 Wu, L. et al. Rational design of core-shell-structured CoP_x@FeOOH for efficient seawater electrolysis. *Appl. Catal. B-Environ.* **294**, 120256 (2021).

4 Yu, L. et al. Non-noble metal-nitride based electrocatalysts for high-performance alkaline seawater electrolysis. *Nat. Commun.* **10**, 5106 (2019).

5 Kuang, Y. et al. Solar-driven, highly sustained splitting of seawater into hydrogen and oxygen fuels. *Proc. Natl. Acad. Sci. U. S. A.* **116**, 6624-6629 (2019).

6 Ma, T. et al. The critical role of additive sulfate for stable alkaline seawater oxidation on nickel-based electrodes. *Angew. Chem. Int. Edit.* **60**, 22740-22744 (2021).

7 Li, P. et al. Common-ion effect triggered highly sustained seawater electrolysis with additional NaCl production. *Research* **2020**, 2872141 (2020).

8 Song, H. J., Yoon, H., Ju, B., Lee, D. & Kim, D. Electrocatalytic selective oxygen evolution of carbon-coated $Na_2Co_{1-x}Fe_xP_2O_7$ nanoparticles for alkaline seawater electrolysis. *ACS Catal.* **10**, 702-709 (2020).

9 Pinho, S. P. & Macedo, E. A. Solubility of NaCl, NaBr, and KCl in water, methanol, ethanol, and their mixed solvents. *J. Chem. Eng. Data* **50**, 29-32 (2004).

10 Wilde, B. E. & Williams, E. The relevance of accelerated electrochemical pitting tests to the long-term pitting and crevice corrosion behavior of stainless steels in marine environments. *J. Electrochem. Soc.* **118**, 1057 (1971).

11 Sharma, S. K. *Green corrosion chemistry and engineering* (Wiley-VCH Verlag GmbH & Co. KGaA, 2011).

12 Soltis, J. Passivity breakdown, pit initiation and propagation of pits in metallic materials - review. *Corros. Sci.* **90**, 5-22 (2015).

13 Burstein, G. T., Liu, C., Souto, R. M. & Vines, S. P. Origins of pitting corrosion. *Corros. Eng. Sci. Techn.* **39**, 25-30 (2013).

14 Pistorius, P. C. & Burstein, G. T. Metastable pitting corrosion of stainless steel and the transition

to stability. *Philos. Trans. R. Soc. Lond. A* **341**, 531-559 (1997).

15 Xu, W. et al. Fast and stable electrochemical eroduction of H_2O_2 by electrode architecture engineering. *ACS Sustain. Chem. Eng.* **9**, 7120-7129 (2021).

16 Jiang, K. et al. Isolated Ni single atoms in graphene nanosheets for high-performance CO_2 reduction. *Energy Environ. Sci.* **11**, 893-903 (2018).

17 Keddam, M., Mottos, O. R. & Takenouti, H. Reaction model for iron dissolution studied by electrode impedance: I . Experimental results and reaction model. *J. Electrochem. Soc.* **128**, 257-266 (1981).

18 Epelboin, I. & Keddam, M. Faradaic impedances: Diffusion impedance and reaction impedance. *J. Electrochem. Soc.* **117**, 1052 (1970).

19 Mamlouk, M. & Scott, K. Analysis of high temperature polymer electrolyte membrane fuel cell electrodes using electrochemical impedance spectroscopy. *Electrochim. Acta* **56**, 5493-5512 (2011).

20 Orazem, M. E. & Tribollet, B. *Electrochemical impedance spectroscopy* (John Wiley & Sons, Inc, 2017).

21 Vivier, V. & Orazem, M. E. Impedance analysis of electrochemical systems. *Chem. Rev.* **122**, 11131-11168 (2022).

22 Amor, Y. B., Sutter, E. M. M., Takenouti, H., Orazem, M. E. & Tribollet, B. Interpretation of electrochemical impedance for corrosion of a coated silver film in terms of a pore-in-pore model. *J. Electrochem. Soc.* **161**, C573-C579 (2014).

23 Orazem, M. E., Pébère, N. & Tribollet, B. Enhanced graphical representation of electrochemical impedance data. *J. Electrochem. Soc.* **153**, B129 (2006).

24 Jorcin, J., Orazem, M. E., Pébère, N. & Tribollet, B. CPE analysis by local electrochemical impedance spectroscopy. *Electrochim. Acta* **51**, 1473-1479 (2006).

25 Frankel, G. S. Pitting corrosion of metals: a review of the critical factors. *J. Electrochem. Soc.* **145**, 2186-2198 (1998).

26 Boehni, H. Breakdown of passivity and localized corrosion processes. *Langmuir* **3**, 924-930 (1987).

27 Oblonsky, L. J. & Devine, T. M. A surface enhanced Raman spectroscopic study of the passive films formed in borate buffer on iron, nickel, chromium and stainless steel. *Corros. Sci.* **37**, 17-

41 (1995).

28 Sridhar, N. & Dunn, D. S. In situ study of salt film stability in simulated pits of nickel by Raman and electrochemical impedance spectroscopies. *J. Electrochem. Soc.* **144**, 4243-4253 (1997).

29 Yan, M. et al. SVET method for characterizing anti-corrosion performance of metal-rich coatings. *Corros. Sci.* **52**, 2636-2642 (2010).
