# Supplementary material for: Concerning the stability of seawater electrolysis: a corrosion mechanism study of halide on Ni-based anode
Source: Nat Commun. 2023 Aug 10;14:4822. doi: 10.1038/s41467-023-40563-9 (PMC10415325; doi:10.1038/s41467-023-40563-9)
Supplement: Supplementary file 3 — Description of Additional Supplementary Files [file 41467_2023_40563_MOESM3_ESM.pdf]

Description of Additional Supplementary Files

File Name: Supplementary Movie 1
Description: Supplementary Movie 1 recorded the corrosion process of Ni foil in ClE, which was taken under the in-situ optical microscope and processed to a 10x playback rate.

File Name: Supplementary Movie 2
Description: Supplementary Movie 2 recorded the corrosion process of Ni foil in BrE , which was taken under the in-situ optical microscope and processed to a 10x playback rate.
